# Supplementary material for: Antibody elicited by HIV-1 immunogen vaccination in macaques displaces Env fusion peptide and destroys a neutralizing epitope
Source: NPJ Vaccines. 2021 Oct 25;6:126. doi: 10.1038/s41541-021-00387-4 (PMC8545924; doi:10.1038/s41541-021-00387-4)
Supplement: Supplementary file 1 — Supplementary Information [file 41541_2021_387_MOESM1_ESM.pdf]

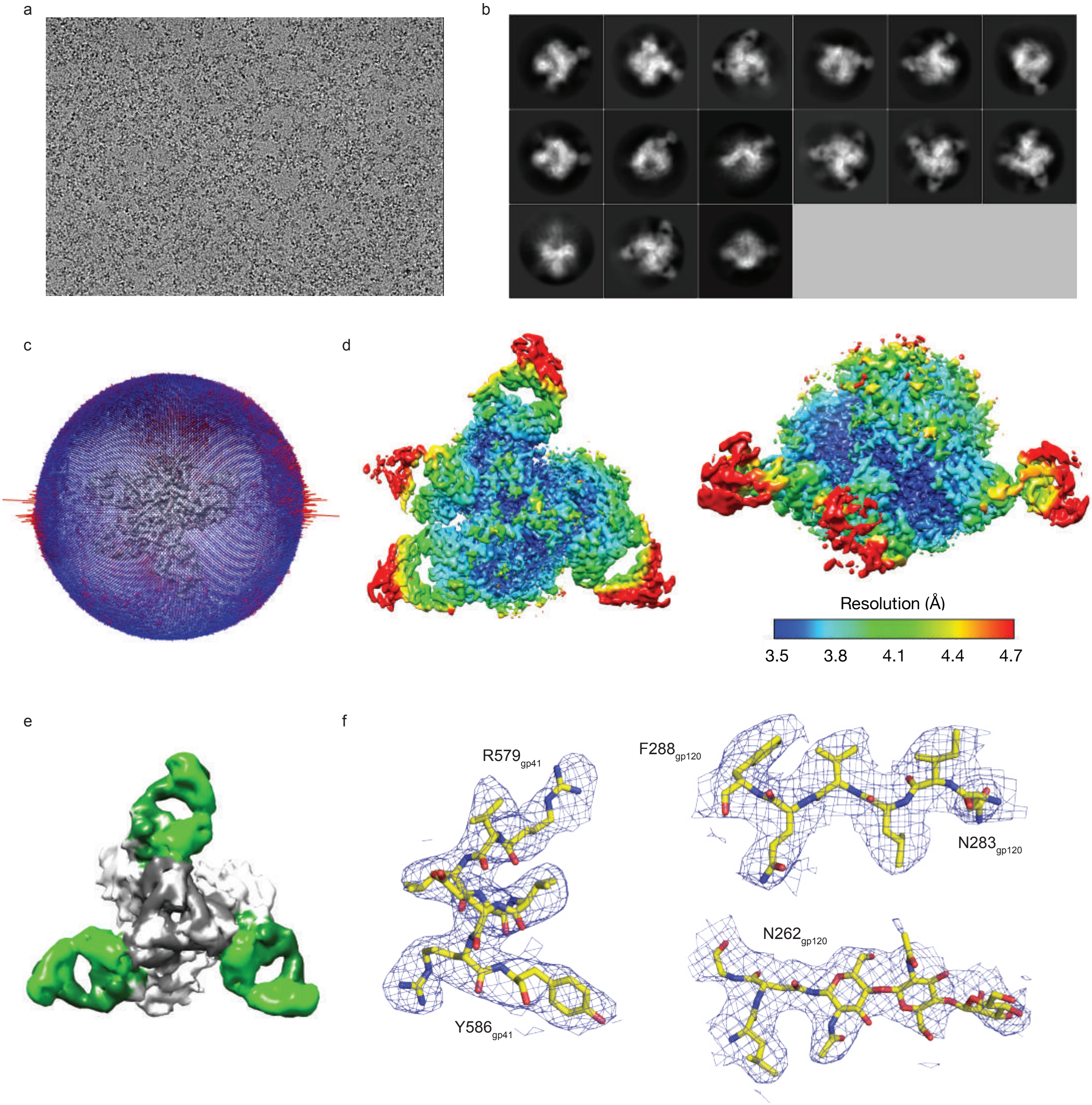

Supplementary Fig. 1. Cryo-EM Data Processing. a, Representative motion-corrected micrograph. **b**, 4x-binned selected 2D classes. **c**, Orientation distribution analysis. **d**, Local resolution map from two angles**. e**, Map of 3D class that contained 3 8ANC195 Fabs and no Ab1245 Fabs (colors as in Fig. 2b). **f**, Representative images of map quality at indicated sites for the 3.7 Å Ab1245–BG505–8ANC195 cryo-EM structure.

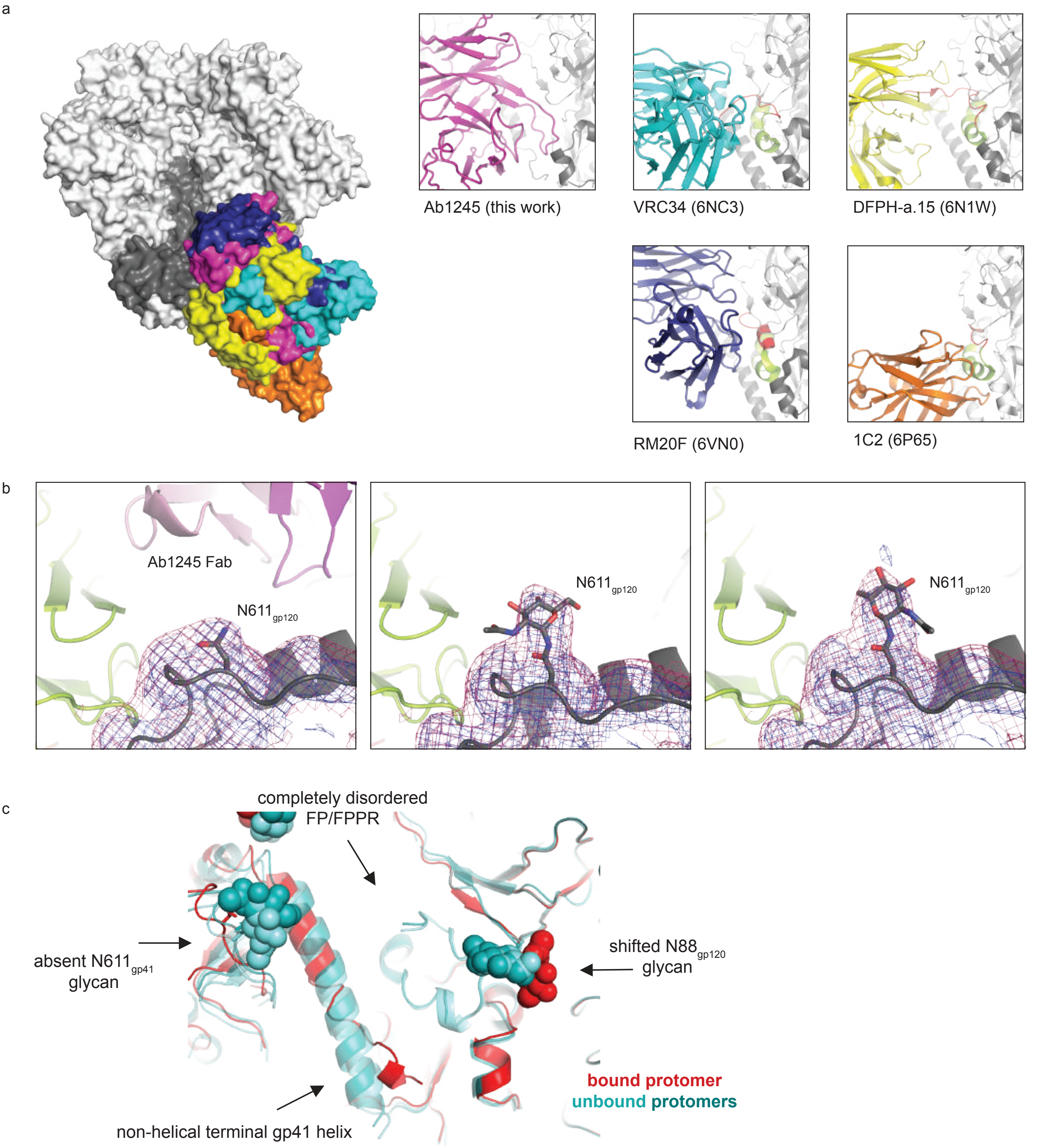

Supplementary Fig. 2. Ab1245 Structural Analysis. a, Comparison of binding of gp120-gp41 interface or fusion peptide-directed antibodies. Left: BG505 trimer (gray surface) and Fab V_H-V_L domains (colored surfaces). Insets: individual antibodies bound to Env trimer (gray) are shown as cartoon with the FP in red and the FPPR in orange. **b**, Comparison of density for the $N611_{gp41}$ glycan on gp120 (gray cartoon), with 8ANC195 (green cartoon), and Ab1245 (pink cartoon with non-blurred (blue) and blurred (red) maps shown as mesh). **c**, Comparison of secondary structure between protomers of gp120-gp41 with Ab1245 bound (red cartoon) and not bound (shades of teal, cartoon). Glycans are shown as spheres, and differences between bound and unbound protomers are highlighted with arrows and descriptions.
