# Supplementary material for: Antibody elicited by HIV-1 immunogen vaccination in macaques displaces Env fusion peptide and destroys a neutralizing epitope
Source: NPJ Vaccines. 2021 Oct 25;6:126. doi: 10.1038/s41541-021-00387-4 (PMC8545924; doi:10.1038/s41541-021-00387-4)
Supplement: Supplementary file 2 — Reporting Summary [file 41541_2021_387_MOESM2_ESM.pdf]

nature research

Corresponding author(s): Pamela Bjorkman

Last updated by author(s): Jun 3, 2021

Reporting Summary

Nature Research wishes to improve the reproducibility of the work that we publish. This form provides structure for consistency and transparency in reporting. For further information on Nature Research policies, see our Editorial Policies and the Editorial Policy Checklist.

Statistics

For all statistical analyses, confirm that the following items are present in the figure legend, table legend, main text, or Methods section.

n/a	Confirmed	
☐	☒	The exact sample size (*n*) for each experimental group/condition, given as a discrete number and unit of measurement
☐	☒	A statement on whether measurements were taken from distinct samples or whether the same sample was measured repeatedly
☒	☐	The statistical test(s) used AND whether they are one- or two-sided<br>*Only common tests should be described solely by name; describe more complex techniques in the Methods section.*
☒	☐	A description of all covariates tested
☒	☐	A description of any assumptions or corrections, such as tests of normality and adjustment for multiple comparisons
☒	☐	A full description of the statistical parameters including central tendency (e.g. means) or other basic estimates (e.g. regression coefficient) AND variation (e.g. standard deviation) or associated estimates of uncertainty (e.g. confidence intervals)
☒	☐	For null hypothesis testing, the test statistic (e.g. *F*, *t*, *r*) with confidence intervals, effect sizes, degrees of freedom and *P* value noted<br>*Give P values as exact values whenever suitable.*
☒	☐	For Bayesian analysis, information on the choice of priors and Markov chain Monte Carlo settings
☒	☐	For hierarchical and complex designs, identification of the appropriate level for tests and full reporting of outcomes
☒	☐	Estimates of effect sizes (e.g. Cohen's *d*, Pearson's *r*), indicating how they were calculated

Our web collection on statistics for biologists contains articles on many of the points above.

Software and code

Policy information about availability of computer code

Data collection	SerialEM v3.7 for micrograph collection. Mass Photometry software AcquireMP 2.2.0.
Data analysis	SPR from Biacore T200 software v3.0. SEC-MALS from ASTRA 6. Mass photometry used DiscoverMP 2.2.0. Phenix v1.15, Coot v0.8.9, RELION v3.0, PyMOL v2.1, and Chimera v1.13 were used for structural analysis.

For manuscripts utilizing custom algorithms or software that are central to the research but not yet described in published literature, software must be made available to editors and reviewers. We strongly encourage code deposition in a community repository (e.g. GitHub). See the Nature Research guidelines for submitting code & software for further information.

Data

Policy information about availability of data

All manuscripts must include a data availability statement. This statement should provide the following information, where applicable:

- Accession codes, unique identifiers, or web links for publicly available datasets
- A list of figures that have associated raw data
- A description of any restrictions on data availability

The atomic model and cryo-EM maps have been deposited in the Protein Data Bank (PDB) accession code 7MXE and Electron Microscopy Data Bank (EMDB) entry EMD-24072.

Field-specific reporting

Please select the one below that is the best fit for your research. If you are not sure, read the appropriate sections before making your selection.

☒ Life sciences ☐ Behavioural & social sciences ☐ Ecological, evolutionary & environmental sciences

For a reference copy of the document with all sections, see nature.com/documents/nr-reporting-summary-flat.pdf

Life sciences study design

All studies must disclose on these points even when the disclosure is negative.

Sample size	No sample size calculation was performed to design this study.
Data exclusions	No data were excluded.
Replication	All experiments were replicated successfully; n values can be found in figure legends or methods.
Randomization	Not applicable to study; no experiments used randomized data.
Blinding	Not applicable to study; no experiments required blinding.

Reporting for specific materials, systems and methods

We require information from authors about some types of materials, experimental systems and methods used in many studies. Here, indicate whether each material, system or method listed is relevant to your study. If you are not sure if a list item applies to your research, read the appropriate section before selecting a response.

Materials & experimental systems

n/a	Involved in the study	
☐	☒	Antibodies
☐	☒	Eukaryotic cell lines
☒	☐	Palaeontology and archaeology
☒	☐	Animals and other organisms
☒	☐	Human research participants
☒	☐	Clinical data
☒	☐	Dual use research of concern

Methods

n/a	Involved in the study	
☒	☐	ChIP-seq
☒	☐	Flow cytometry
☒	☐	MRI-based neuroimaging

Antibodies

Antibodies used	The following anti-human antibody cocktail was used for sorting: anti-CD16 APC-eFluor780 (Invitrogen, #47-0168-41), anti-CD8[illegible] APC-eFluor780 (Invitrogen, #47-0086-42), anti-CD3 APC-eFluor780 (Invitrogen, #47-0037-41), anti-CD14 APC-eFluor780 (eBiosciences, #47-0149-41), anti-CD20 PeCy7 (BD, #335793), anti-CD38 FITC (Stem Cell technologies, #60131FI) at a 1:200 dilution. Ab1245 was reported in this study.
Validation	All of the listed antibodies are commercially available and have been validated by their respective manufacturers.

Eukaryotic cell lines

Policy information about cell lines

Cell line source(s)	Expi293F cells (Thermo Fisher)
Authentication	Cell line not authenticated after purchase
Mycoplasma contamination	The cell lines were not contaminated by mycoplasma as determined by using the Lonza Mycoplasma Detection Kit.
Commonly misidentified lines (See ICLAC register)	No commonly misidentified cell lines were used.
